# Cavernous Hemangioma of Temporalis Muscle: A Case Report

**DOI:** 10.29252/wjps.9.1.99

**Published:** 2020-01

**Authors:** Gholamreza Motazedian, Ali Khojasteh, Nasrin Motazedian, Mohammad Hossein Anbardar

**Affiliations:** 1 Burn and Wound Healing Research Center, Plastic and Reconstructive Surgery Ward, Shiraz University of Medical Sciences, Shiraz, Iran;; 2 Shiraz Transplant Research Center, Shiraz University of Medical Sciences, Shiraz, Iran;; 3 Department of Pathology, Namazee Hospital, Shiraz University of Medical Sciences, Shiraz, Iran

**Keywords:** Cavernous hemangioma, Temporalis muscle, Iran

## Abstract

Cavernous hemangioma is an encapsulated nodular mass composed of dilated, cavernous vascular space separated by connective tissue stroma. Flattened endothelial cells line the vascular spaces, which were filled with blood. Though hemangiomas are the mast common benign neoplasms seen in children, they rarely occur in adults. In the head and neck region, the masseter and trapezius muscles are most commonly involved. Herein, the case is a 64 years old male who presented with a round, painless mass in the right temporal fossa with extension to infratemporal fossa. The lesion was surgically excised and histopathology confirmed the diagnosis of cavernous hemangioma.

## INTRODUCTION

Hemangiomas are benign tumors with vascular origin and three types of hemangioma have been described according to the vessel type involved: cavernous, capillary and mixed.^1^^,^^2^ Intramuscular hemangiomas make up less than 1% of all hemangiomas and are mostly located in the extremities and the trunk.^3^^,^^4^ Hemangiomas of the head and neck make up less than 15% of intramuscular hemangiomas.^3^^,^^5^ We present a patient with cavernous hemangioma located in the temporalis muscle with extension to infratemporal fossa.

## CASE REPORT

**Fig. 1:**
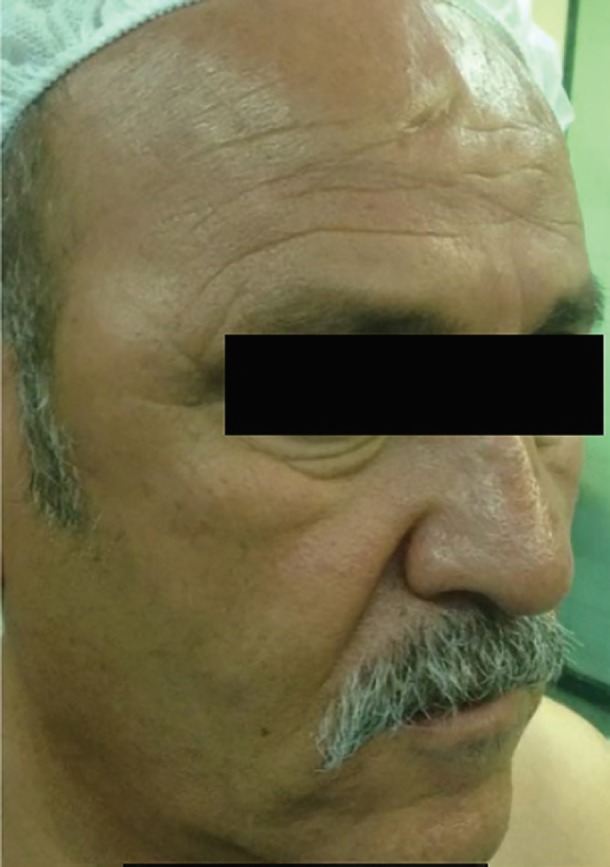
Initial photography of the patient showed swelling of right temporal area and infraorbital region

**Fig. 2:**
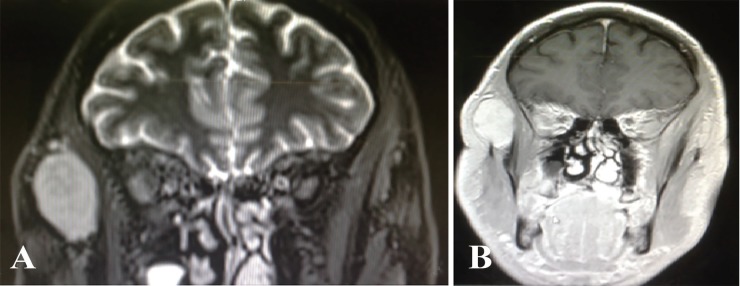
MRI shows a large soft tissue mass within the right temporalis muscle with extension to right infratemporal fossa. (A): The tumor has isointense signal on T1 weighted sequences. (B): The tumor has a high signal on T2 weighted sequences

A 64-year old man with painless, soft right temporal swelling and right infraorbital edema that had been noticed by himself several months ago has been presented. Physical examination showed 4×5 cm round and painless mass situated lateral to orbital wall. His problem was associated with right infraorbital edema. Fine needle aspiration was not diagnostic (Figure 1). MRI showed a large soft tissue mass within the right temporalis muscle with extension to right infratemporal fossa. The tumor had isointense signal on T1 weighted sequences and a high signal on T2 weighted sequences (Figure 2).

**Fig. 3:**
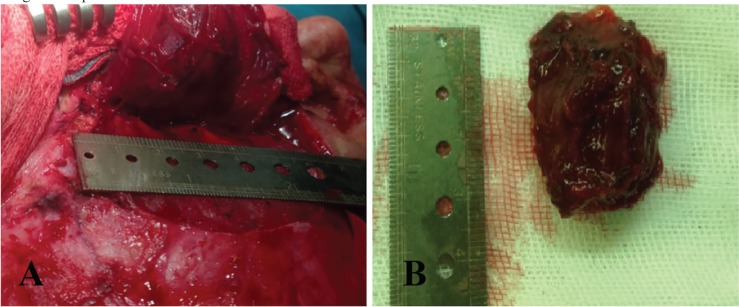
(A): Intra-operative view of the hemangioma approached through coronal incision. (B): Surgical specimen. Mass was well circumscribed and excised with a margin of normal mu

**Fig. 4:**
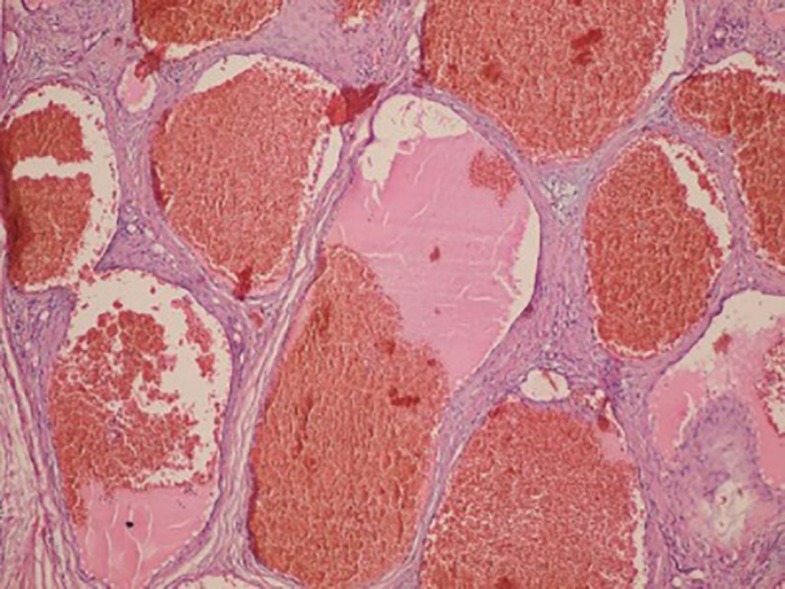
Section shows dilated blood vessels arranged back to back and containing blood and lymph (Hematoxylin and Eosin, ×100).

Surgical excision was performed through coronal incision. The mass was found within the temporal muscle and was excised with loupe magnification to protect right frontal branch of facial nerve. Blood loss during operation was trace. Postoperative recovery was uneventful and functional and cosmetic results were excellent (Figure 3). Gross examination of mass showed a rubbery round tissue with measurement of 3.5×2.8×2 cm, and cut sections showed dark red brown surface with microcytic appearance. Histological study showed an ill-defined mass consisted of dilated channel-like blood vessels, containing lymph and blood, which were entrapped between muscles. There was no evidence of atypia, necrosis and mitosis. The diagnosis of intramuscular cavernous hemangioma was signed out for him. Postoperative follow up during 1-year period showed no recurrence (Figure 4).

## DISCUSSION

Hemangiomas are benign vascular proliferations and one of the commonest benign soft tissue tumors consisting 7% of all soft tissue tumors. They are usually present in infants and children, while females are more affected than males. The etiology is unclear. Intramuscular hemangiomas are rare tumors accounting for 0.8% of all hemangiomas and approximately 14% of cases are localized in the musculature of the head and neck.^6^ The masseter (36%), followed by the trapezious (24%) are the muscles most frequently affected, whereas temporalis muscle hemangioma are extremely rare.^7^

In 1972, Allen and Enzinger classified intramuscular hemangioma according to the vessel size as (i) capillary type (small vessel), (ii) large vessel or cavernous type, and (iii) mixed type (consisting of both).^1^ The most common presentation is a smooth, non-tender and mobile mass. The lesion usually does not show any of the vascular signs such as thrill or skin discoloration like superficial hemangioma.^8^ Plain x-ray films, CT scan, angiography may not be specific for this tumor. CT scan is helpful for evaluation of bone involvement. MRI is the method of choice. On MRI, the tumor is isointense on T1 and hyperintense on T2 weighted sequences.^9^

Treatment options are variable depending on the size, vascularization and location.^10^ Preoperative embolization can help to reduce intra-operative bleeding.^11^ Sclerosing agents, corticosteroids and radiotherapy are another modalities for treatment.^12^ But, in this case; embolization was not performed due to relatively slow and small amount of blood pooling. Surgical excision is the treatment of choice. Coronal incision is the best way to approach this tumor and the tumor should be excised with loupe magnification to protect frontal branch of facial nerve and securely ligate feeding vessels. Long term clinical and radiological follow up are necessary to detect any relapse.^13^

Hemangiomas are benign vascular tumors and are rarely seen in the temporalis muscle. This case showed a cavernous intramuscular hemangioma involving temporalis muscle in old adult. Surgery is the treatment of choice to exclude malignancy and for adequate treatment of these lesions. Coronal incision is the best way to approach this tumor and the tumor should be excised with loupe magnification to protect frontal branch of facial nerve and securely ligate feeding vessels. Long term clinical and radiological follow up are necessary to detect any relapse.
